# Supplementary material for: Germline polymorphisms and alternative splicing of human immunoglobulin light chain genes
Source: iScience. 2021 Sep 29;24(10):103192. doi: 10.1016/j.isci.2021.103192 (PMC8517844; doi:10.1016/j.isci.2021.103192)
Supplement: Document S1. Figures S1–S8 and Tables S1, S2 [file mmc1.pdf]

iScience, Volume *24*

Supplemental information

Germline polymorphisms and alternative splicing of human immunoglobulin light chain genes

Ivana Mikocziova, Ayelet Peres, Moriah Gidoni, Victor Greiff, Gur Yaari, and Ludvig M. Sollid

Supplemental Table S1. Validation primer sequences. Related to Figure 1 and Figure 2. The primers below were custom-designed using PrimerBLAST. Primers for IGLV2-14, IGLV3-25, IGLV6-57. IGLV7-46 and IGLV10-54 were taken from Vázquez Bernat et al. (2019).

**IGLV1-44_Fwd**	TCAGGGCTCACAACTGTGTT
**IGLV1-44_Rev**	GACCCTGTGTCCTAAGCTGC
**IGKV1-5_Fwd**	TGCATGTTCCCAGAGCACAA
**IGKV1-5_Rev**	AGTCCAGCTGAAGCCATAAAC
**IGKV1-8_Fwd**	TCCAAATAATCCCCATGTGCCA
**IGKV1-8_Rev**	TCCCCCTCTACCAACACCAT
**IGKV4-1_Fwd**	TTCTACGATGCACAAGGCGT
**IGKV4-1_Rev**	CCCAACACACAGAGGAAGCA
Primers from Vázquez Bernat et al. (2019):	
**IGLV2-14_F**	CAGCTGAGCTCCACTATCCAAGGAAGC
**IGLV2-14_R**	GGCCGCACTGCAGGAAGCCGCTTGC
**IGLV3-25_F**	CTCCAAGAGGAGTCCCAGAGGAAG
**IGLV3-25_R**	GGGTGAATTTGGCCAAGCCTACTC
**IGLV6-57_F**	CCCAGATCCTCTGCTGTTTTCCCTC
**IGLV6-57_R**	GGGTAGAGGCCAGAGATGCAAGTC
**IGLV7-46_F**	GAGCAGCTGCTTCCTCCCACAGGAC
**IGLV7-46_R**	CCCCTTTCTATGGCTGGTCCAC
**IGLV10-54_F**	CCTCTCTGCTTCAGATCTTTGAGTAACCAGCAC
**IGLV10-54_R**	CAGTGCTCTGTGAGGCCTGTTCAG

Supplemental Table S2. Sanger sequencing validation results. Related to Figure 1 and Figure 2.

Validated	Not validated
IGLV1-44*01_C146G	IGLV1-44*01_A207C[1‡]
IGLV1-44*01_C215T	IGKV4-1*01_G72A
IGLV2-14*01_T213C_T214C_A220G	IGLV3-25*03_G74C
IGLV6-57*03_C21G	
IGLV7-46*01_A213C	
IGLV10-54*01_A228C	
IGKV1-5*03_G171A	
IGKV1-5*03_T115C_G171A	
IGKV1-8*01_T31C	
IGKV1-8*01_G12A_T31C	
IGKV4-1*01_A318G	

[1‡] Due to its mismatch frequency pattern (see Fig. 2), it is very likely that this inferred polymorphism is in fact a sequencing artefact.

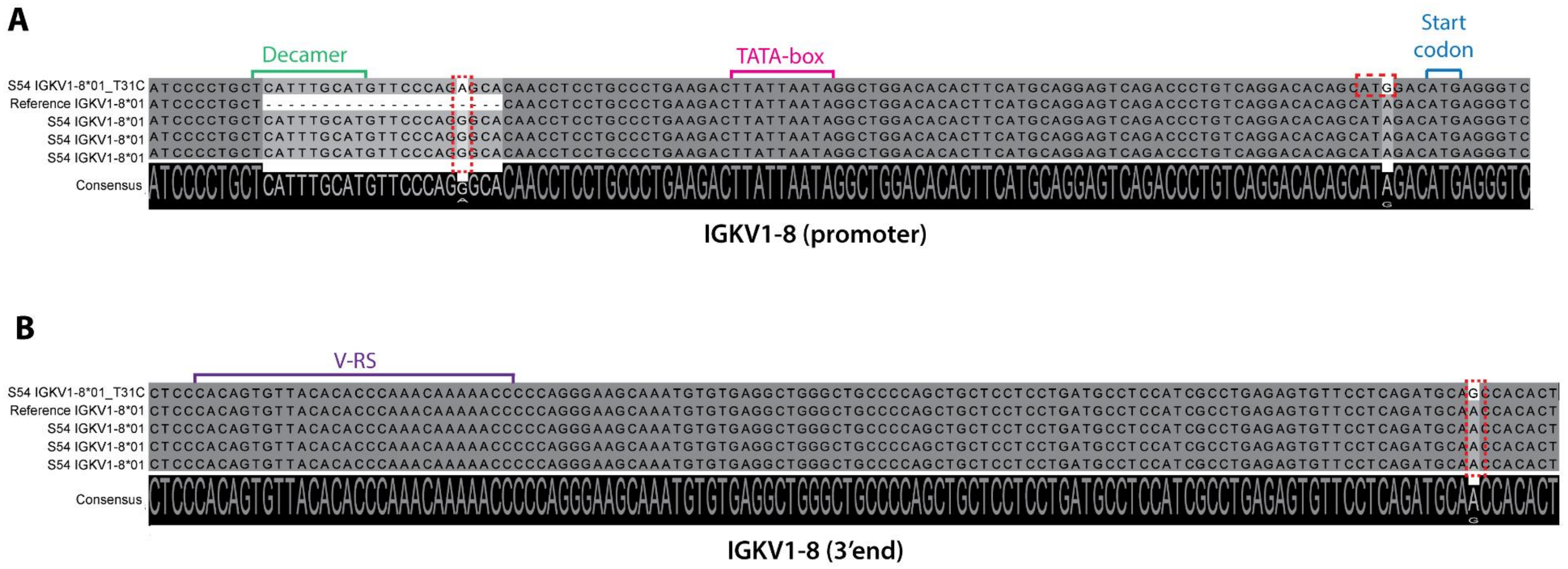

Supplemental Figure S1. Polymorphisms in IGKV1-8 outside of the V-REGION. Related to Figure 1 and Figure 2. Sequences obtained by Sanger sequencing were aligned to the reference sequence of the closest allele available in IMGT/GENE-DB. These alignments were inspected for polymorphisms. (A) The reference sequence for IGKV1-8*01 in the IMGT/GENE-DB appears to be missing 21 nt in the promoter. The novel allele IGKV1-8*01_T31C was found to have additional polymorphisms, highlighted in red, in the promoter, 5'UTR and (B) at the 3'end downstream of the V-RS.

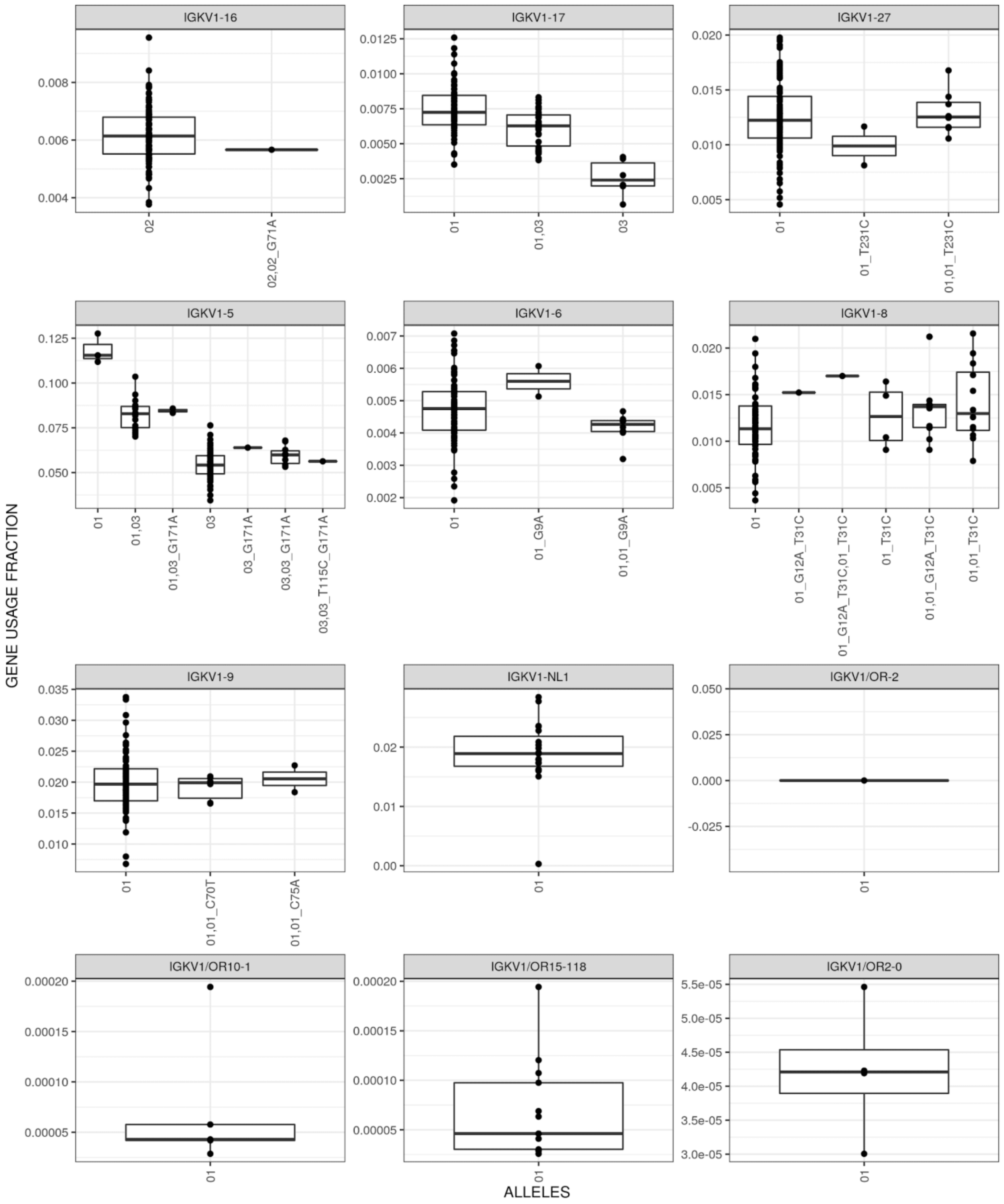

Supplemental Figure S2. Usage of light chain loci genes across individuals in the cohort. Related to Figure 3 and Figure 4. For each individual we calculated the relative usage of the genotyped alleles of each gene. The x-axis shows the inferred alleles, or multiple alleles, that were present in an individual's genotype. Each dot represents a single individual. The y-axis shows the relative usage of an allele or set of alleles for a given gene within the expressed repertoire. The bar represents the median value. The figure continues on the next 7 pages.

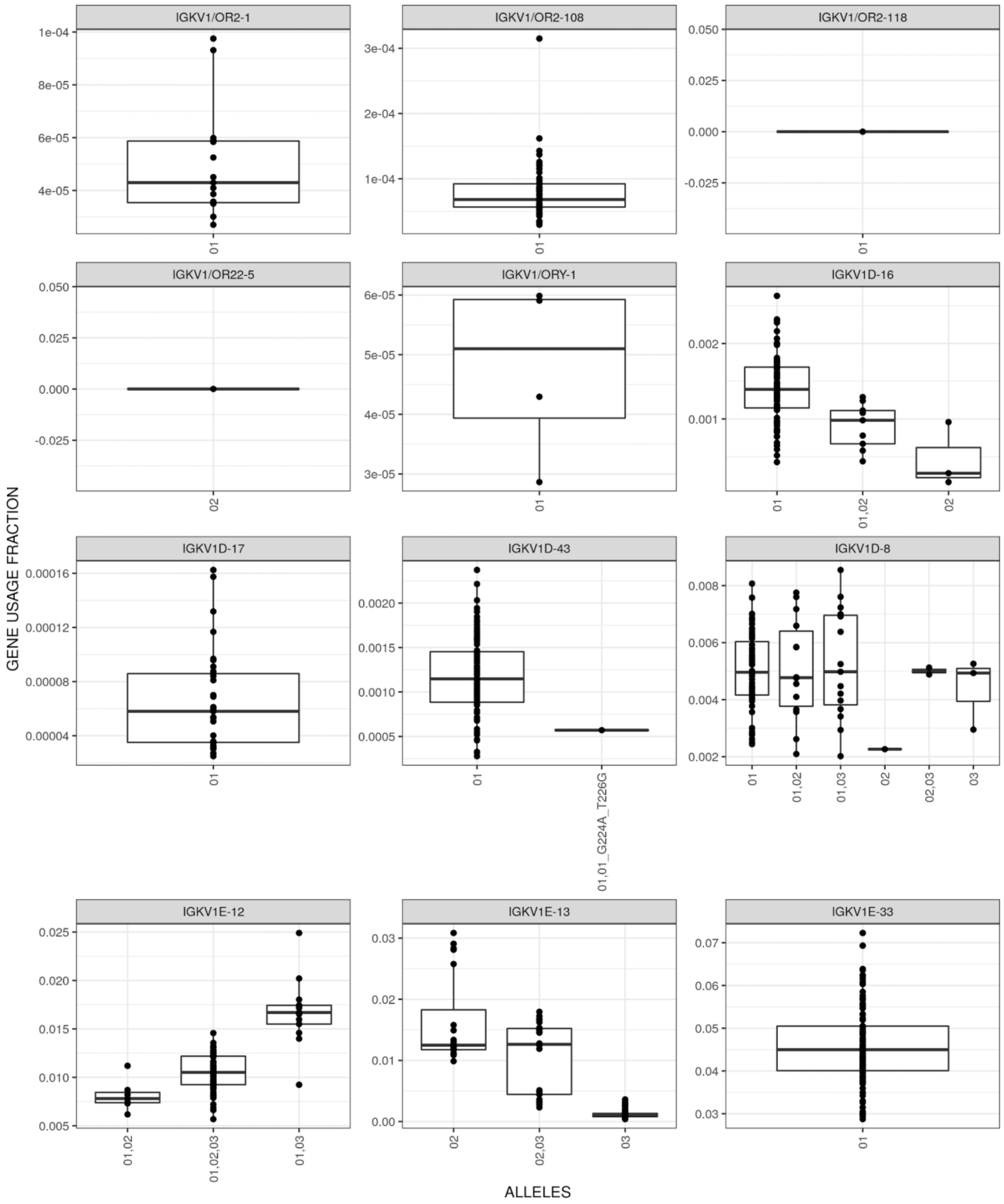

Supplemental Figure S2. continued

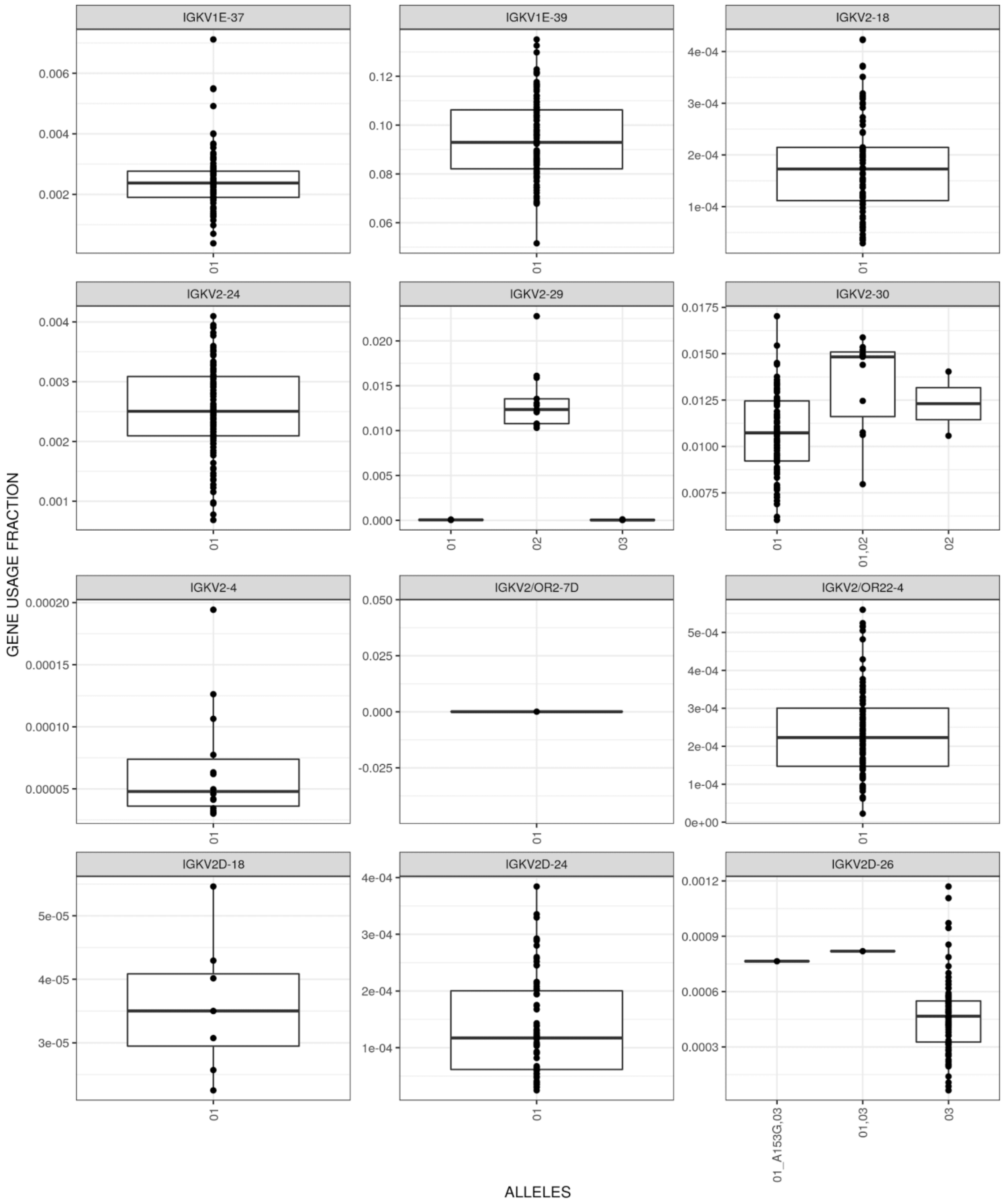

Supplemental Figure S2. continued

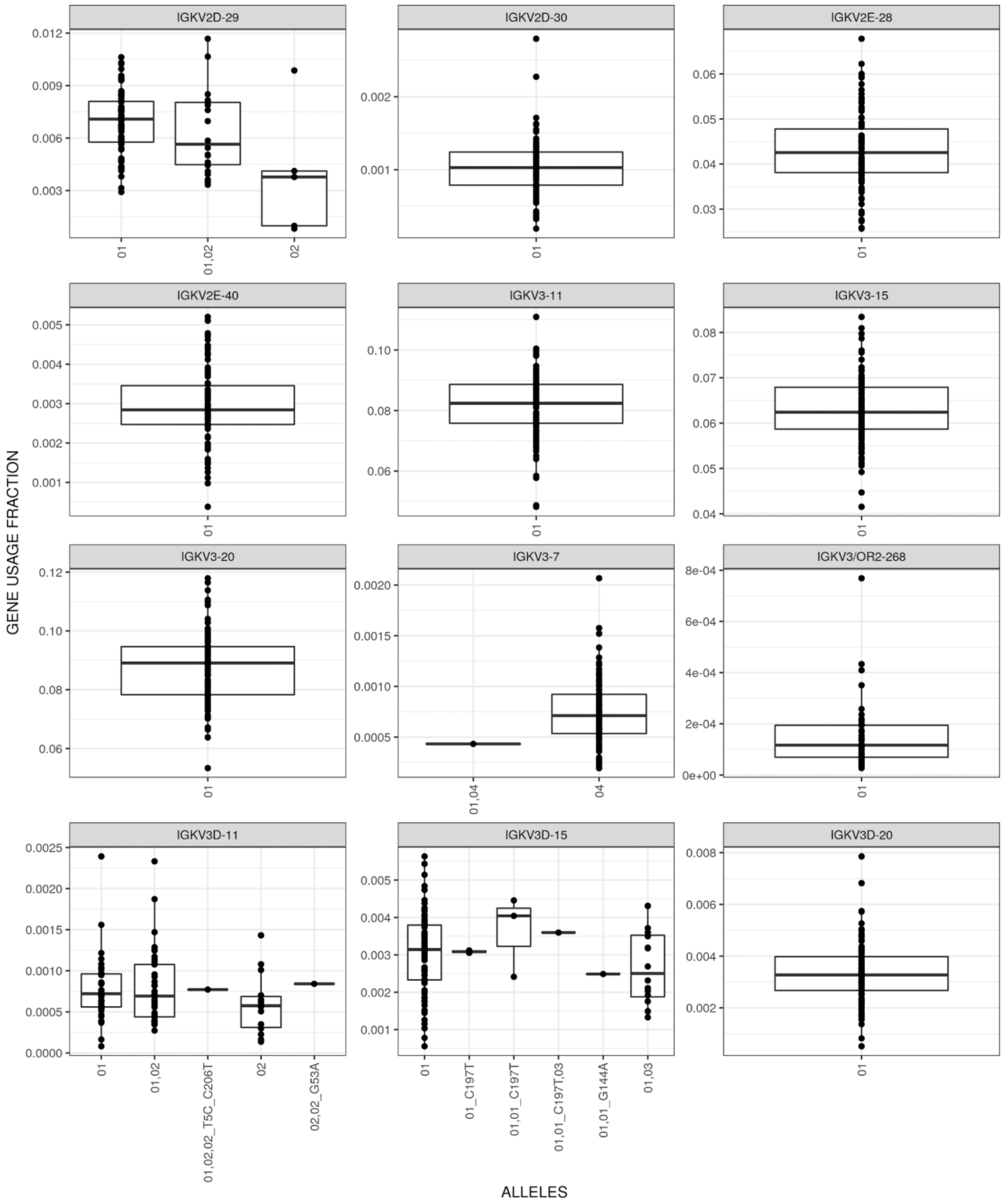

Supplemental Figure S2. continued

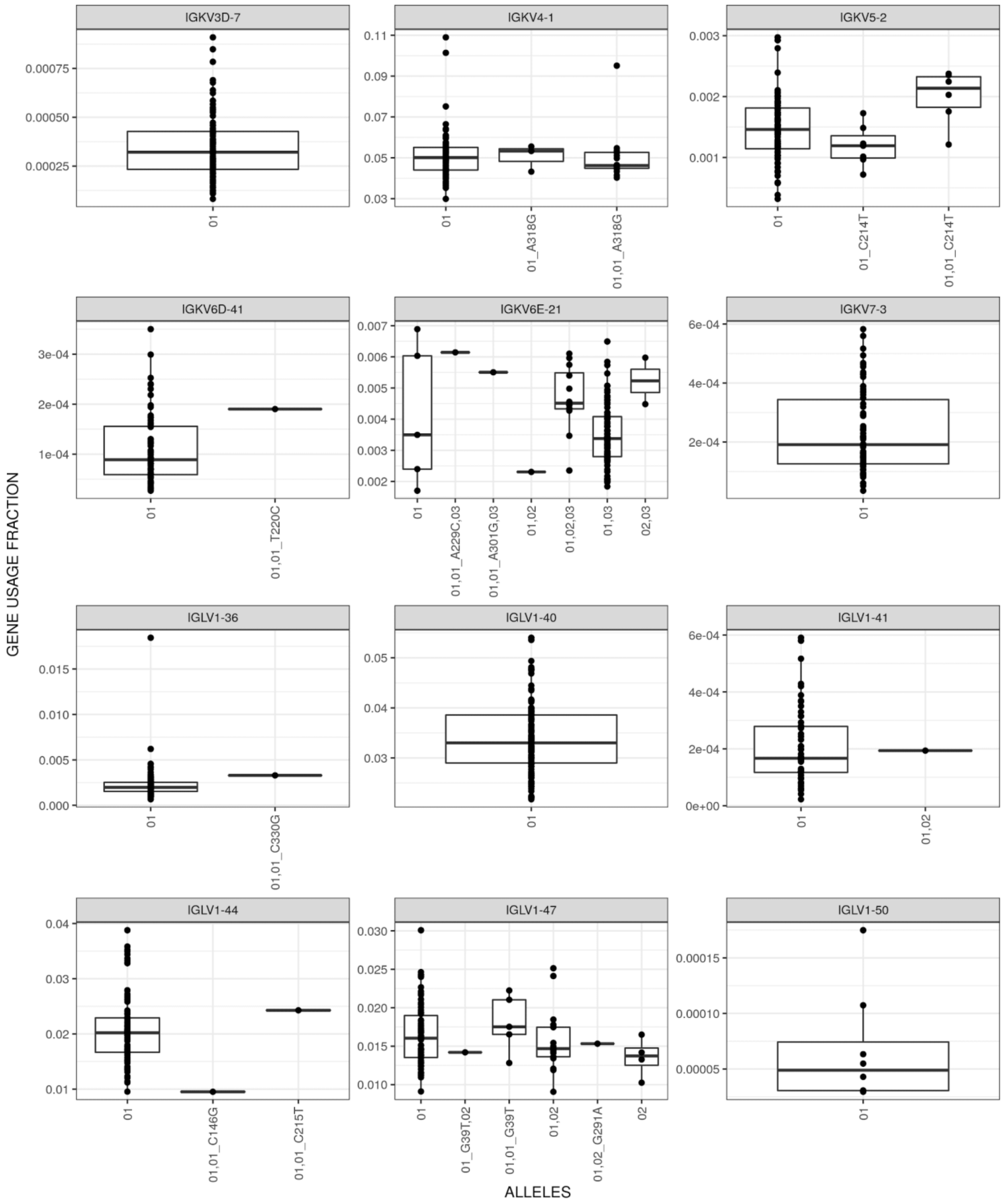

Supplemental Figure S2. continued

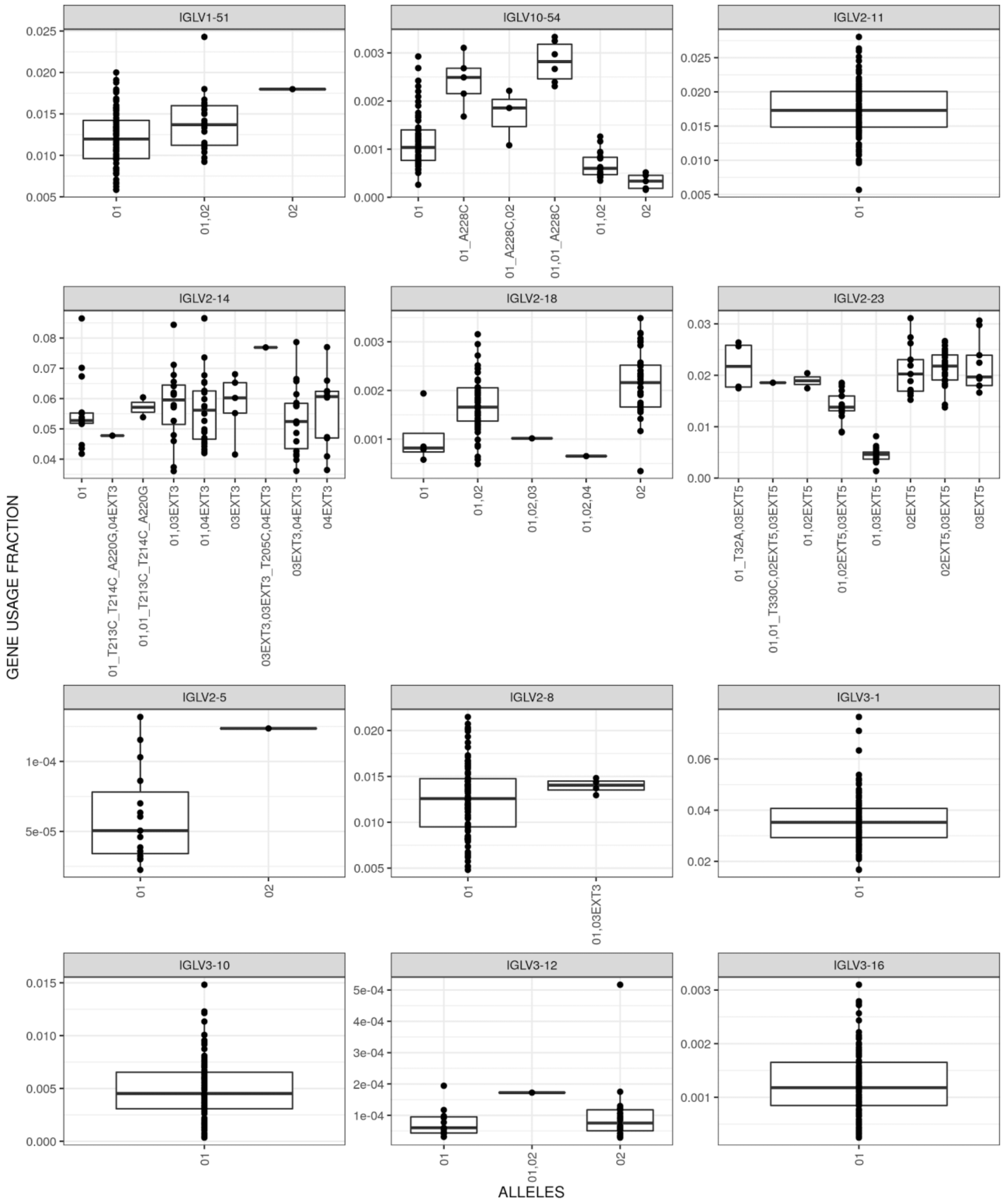

Supplemental Figure S2. continued

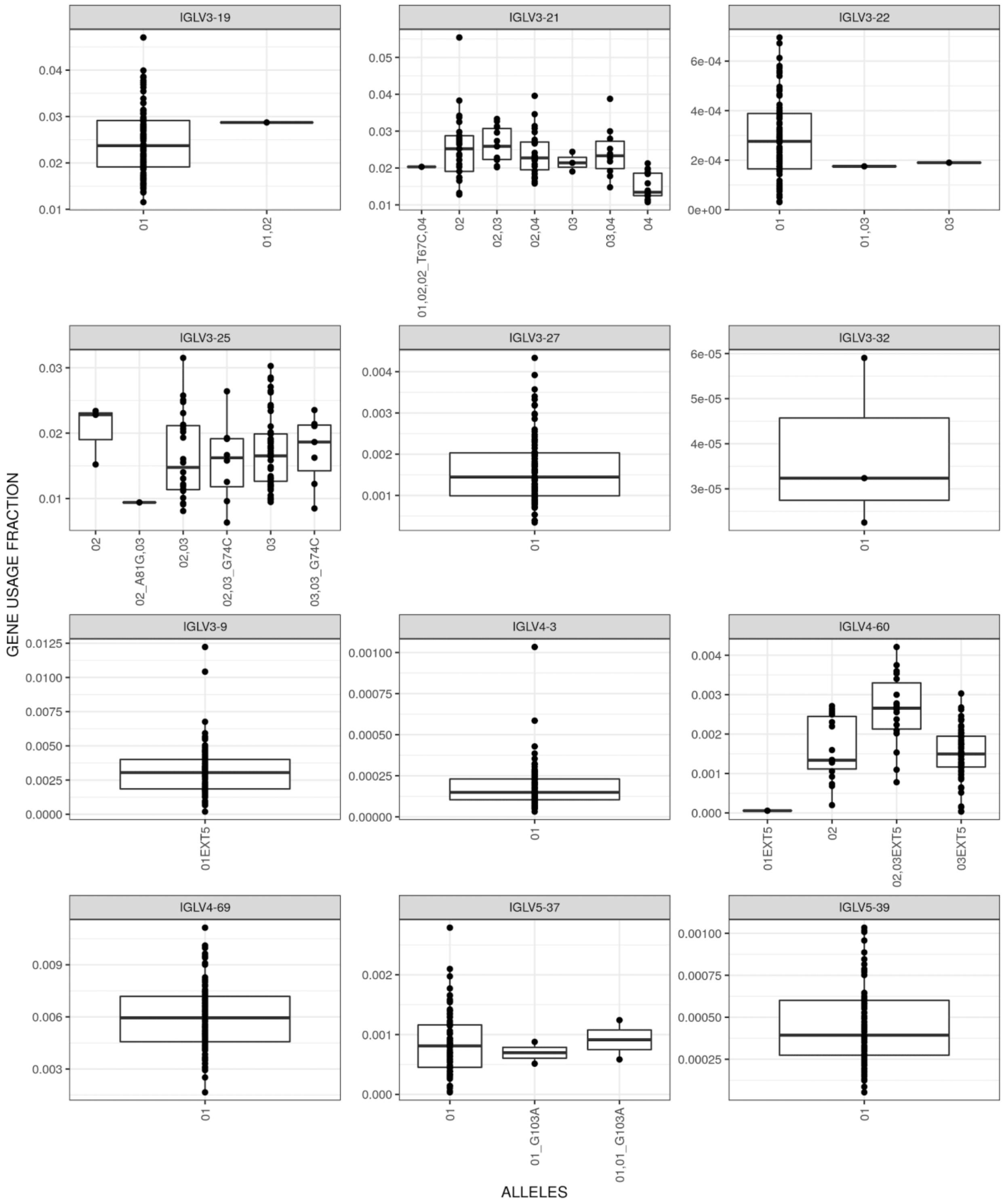

Supplemental Figure S2. continued

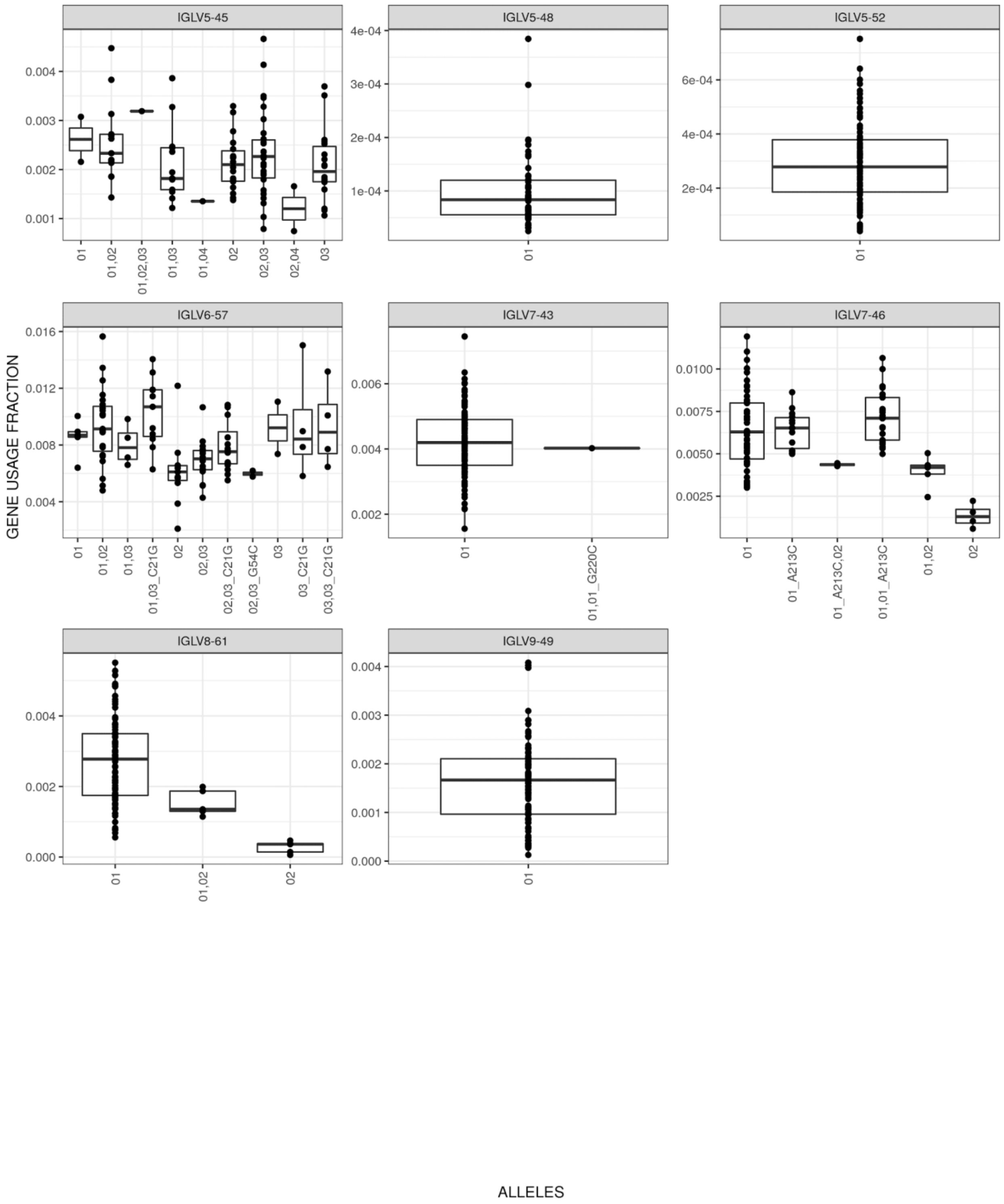

Supplemental Figure S2. continued

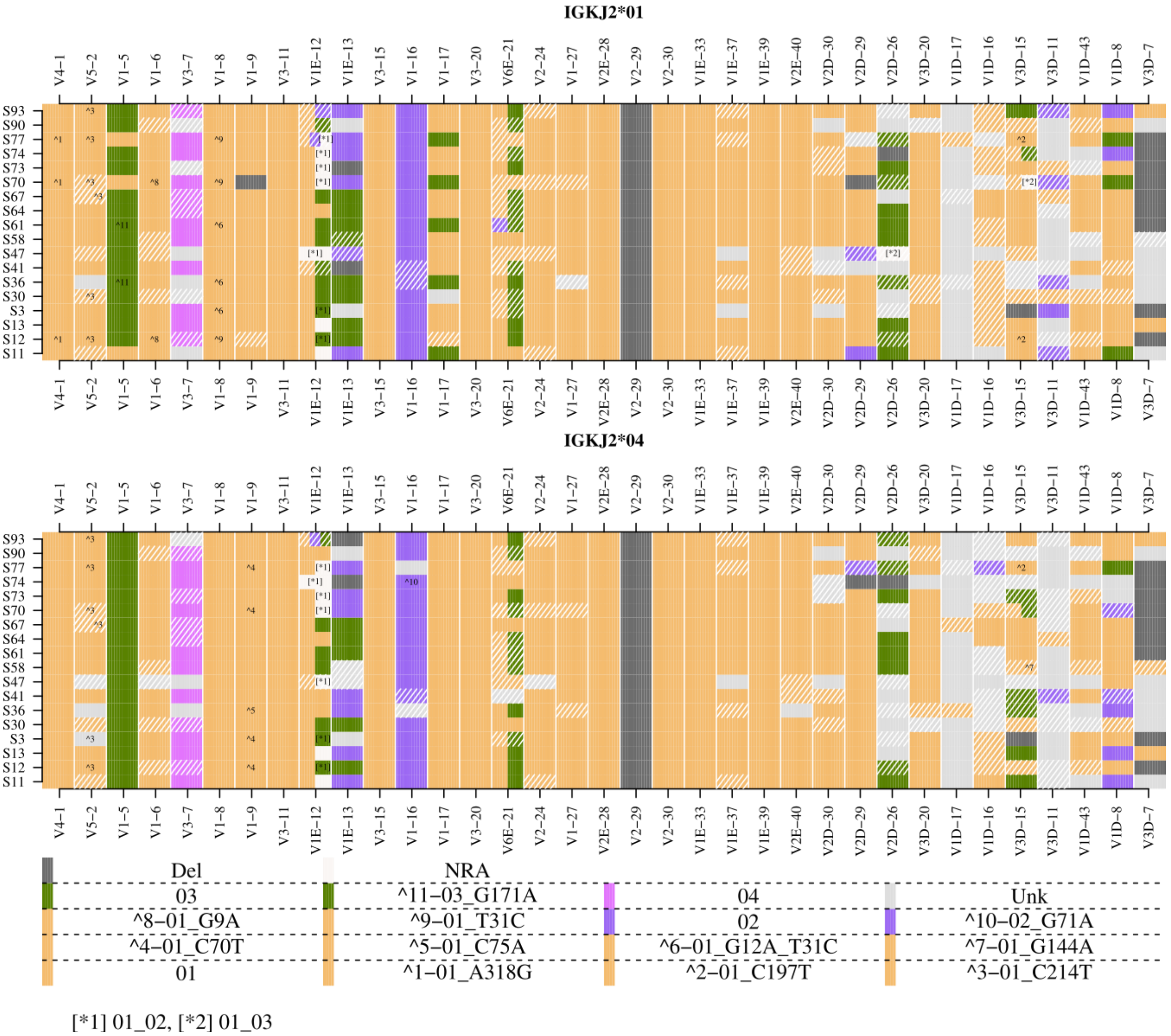

Supplemental Figure S3. Haplotype inference using IGKJ2. Related to Figure 5. To gain more information on the germline variations, we inferred haplotype using the anchor gene IGKJ2. A quarter of our cohort are heterozygous for this gene in a sufficient fraction, a ratio of at least 30:70 between alleles. IGKJ2 has four alleles, and tend to appear in heterozygous from with the alleles *01/*04 (18 individuals) and *01/*03 (6 individuals). Using RAbHIT package we inferred haplotypes with IGKJ2*01/*04. Each panel shows the haplotype inference for a single chromosome, each column is a IGKV gene (x-axis) and each row is an individual (y-axis). The colors represent the different alleles, dark gray represents a chromosomal deletion and light gray an “unknown” call. The white lines show a low certainty level in the inference (lk<1). Novel alleles are annotated in a caret and colored the same as the original alleles. In cases where a gene only appeared in a multiple allele assignment form on a single chromosome, the alleles were collapsed to show that no separation could be made. These cases were annotated with an asterisk and the collapsed allele version.

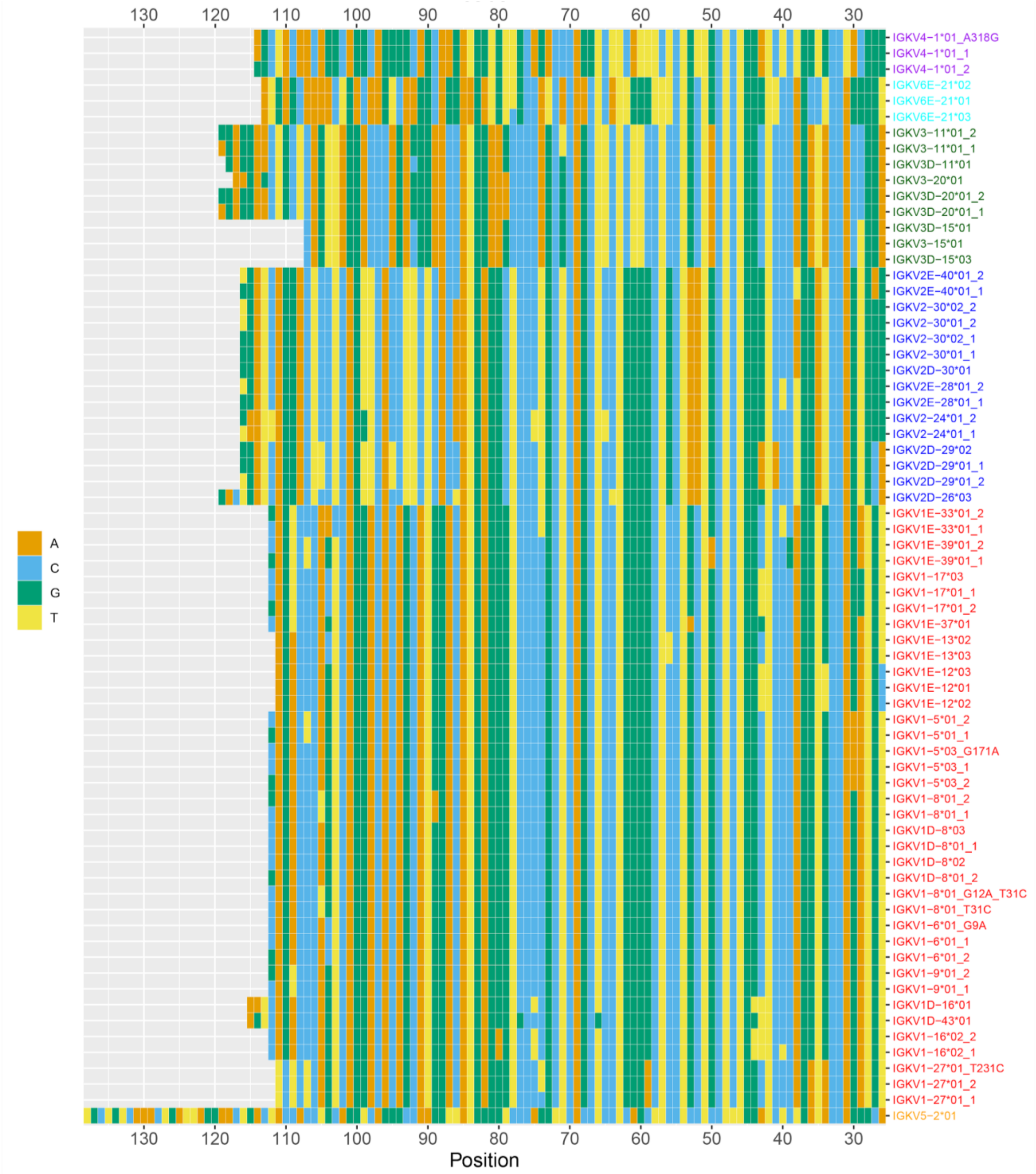

Supplemental Figure S4. Overview of upstream variants of kappa light chain V genes in the cohort. Related to Figure 6. Clustering of upstream sequences of light chain V genes revealed different upstream variants for sequences with the same V-REGION annotation. Position 0 represents the start of the V-REGION and the nucleotides upstream were numbered in 3’ to 5’ direction. The tile colors represent different nucleotides – A is orange, C is blue, G is green and T is yellow. Kappa and lambda upstream variants were plotted separately, upstream variants of the lambda V genes are plotted in Supplemental Figure S5.

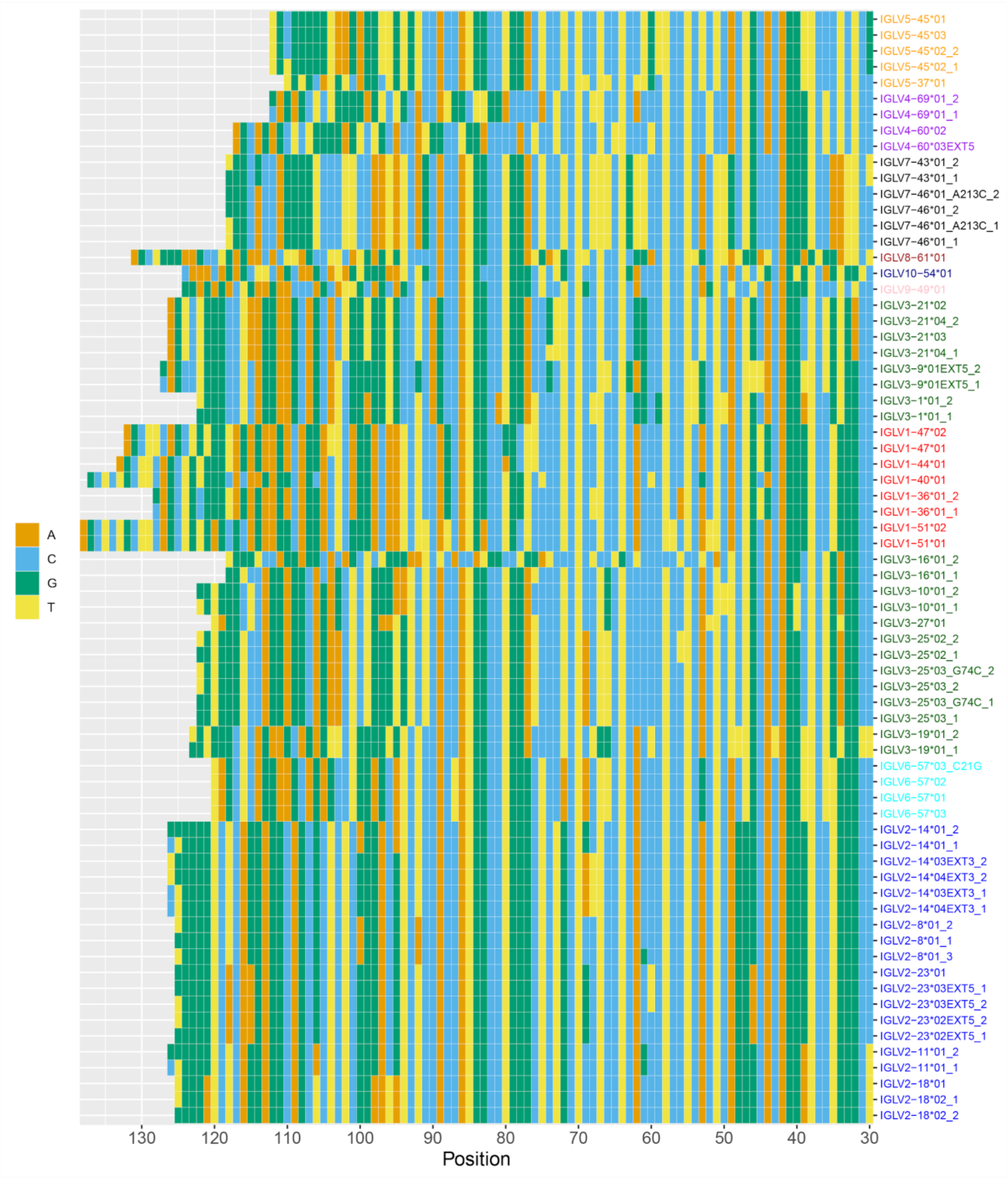

Supplemental Figure S5. Overview of upstream variants of lambda light chain V genes in the cohort. Related to Figure 6. Clustering of upstream sequences of light chain V genes revealed different upstream variants for sequences with the same V-REGION annotation. Position 0 represents the start of the V-REGION and the nucleotides upstream were numbered in 3' to 5' direction. The tile colors represent different nucleotides – A is orange, C is blue, G is green and T is yellow. Kappa and lambda upstream variants were plotted separately, upstream variants of the kappa V genes are plotted in Supplemental Figure S4.

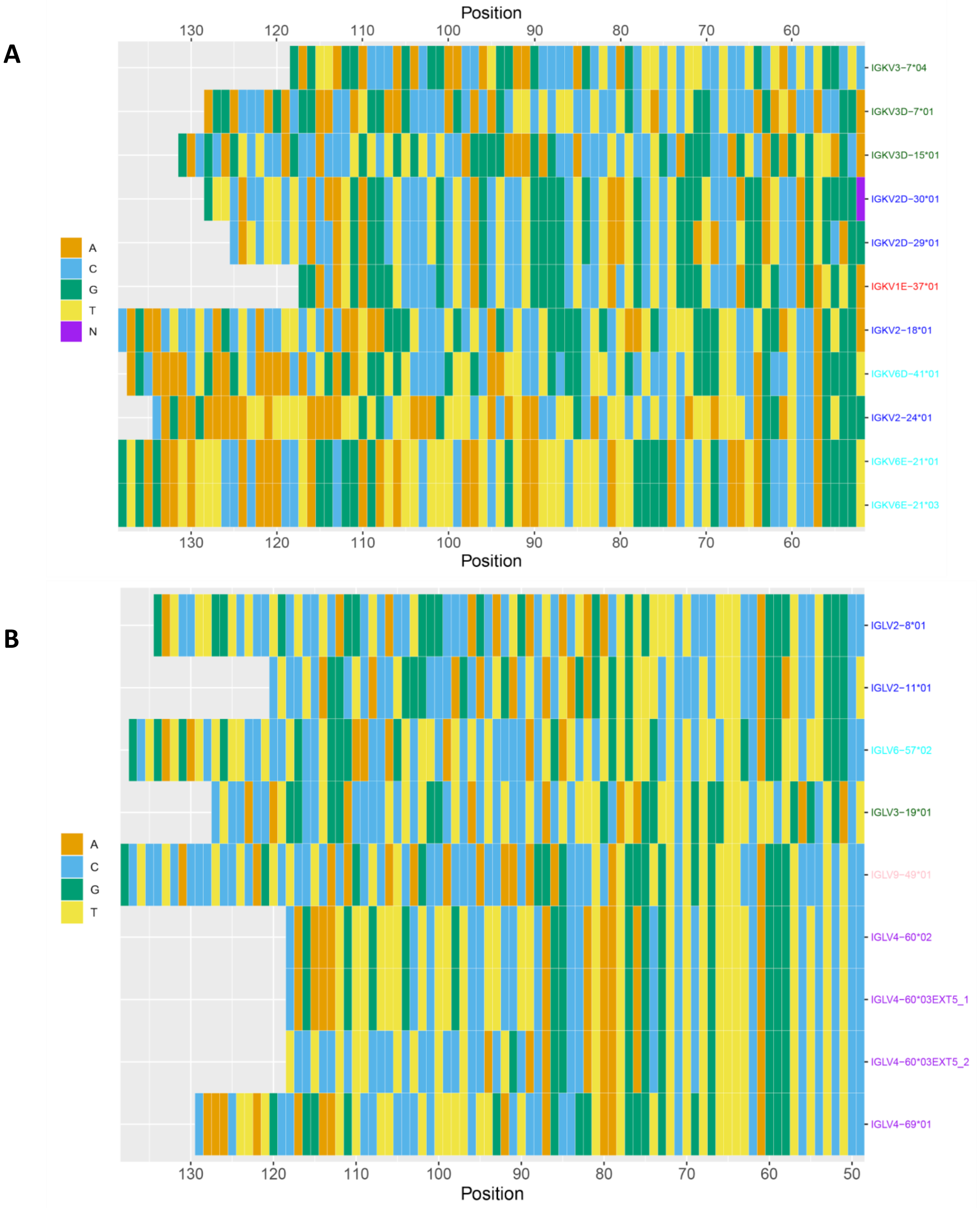

Supplemental Figure S6. Upstream sequences of alternatively spliced transcripts. Related to Figure 6. Several alternatively spliced transcripts were observed by clustering of upstream sequences. The

position numbering is the same as in Supplemental Figure S4. The tile colors represent different nucleotides. Kappa (A) and lambda (B) were analysed separately.

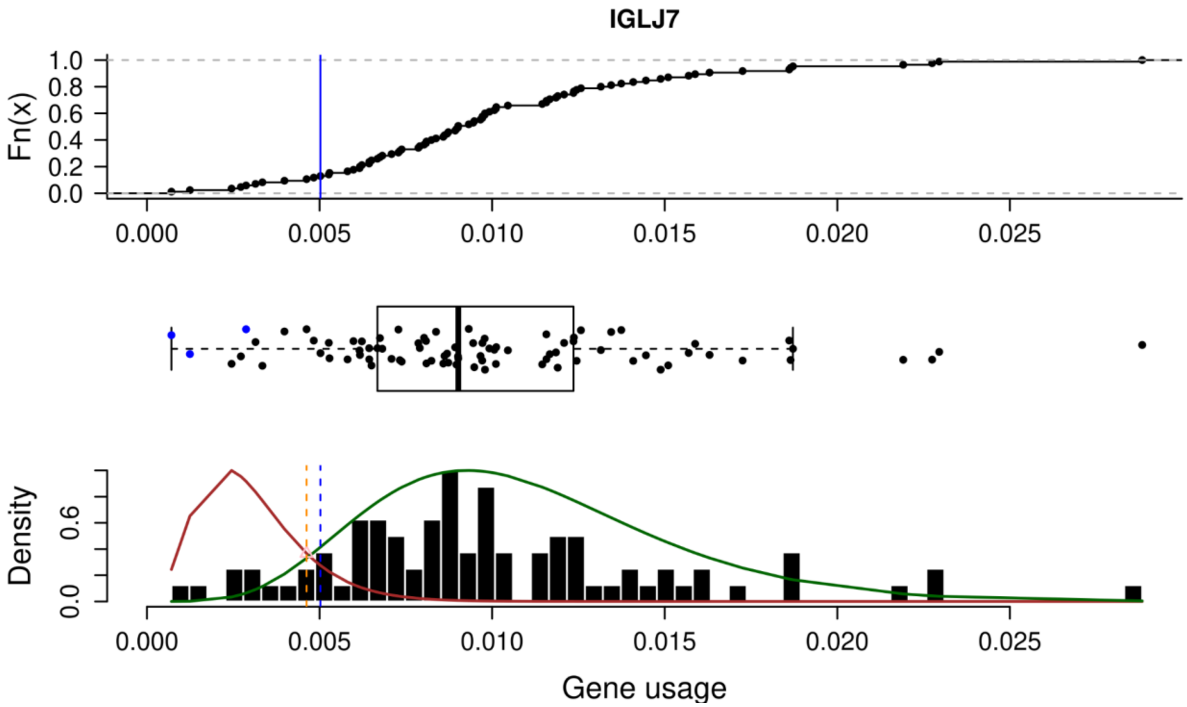

Supplemental Figure S7. Bimodal distribution of IGLJ7. Related to Figure 3. This figure provides additional details to the data shown in Fig.3.The upper panel is the empirical cumulative distribution function curve of the gene usage. The middle panel is a boxplot of the gene usage where the deletions are marked in blue. The lower panel is a histogram of IGLJ7 usage with the estimated gamma distributions.

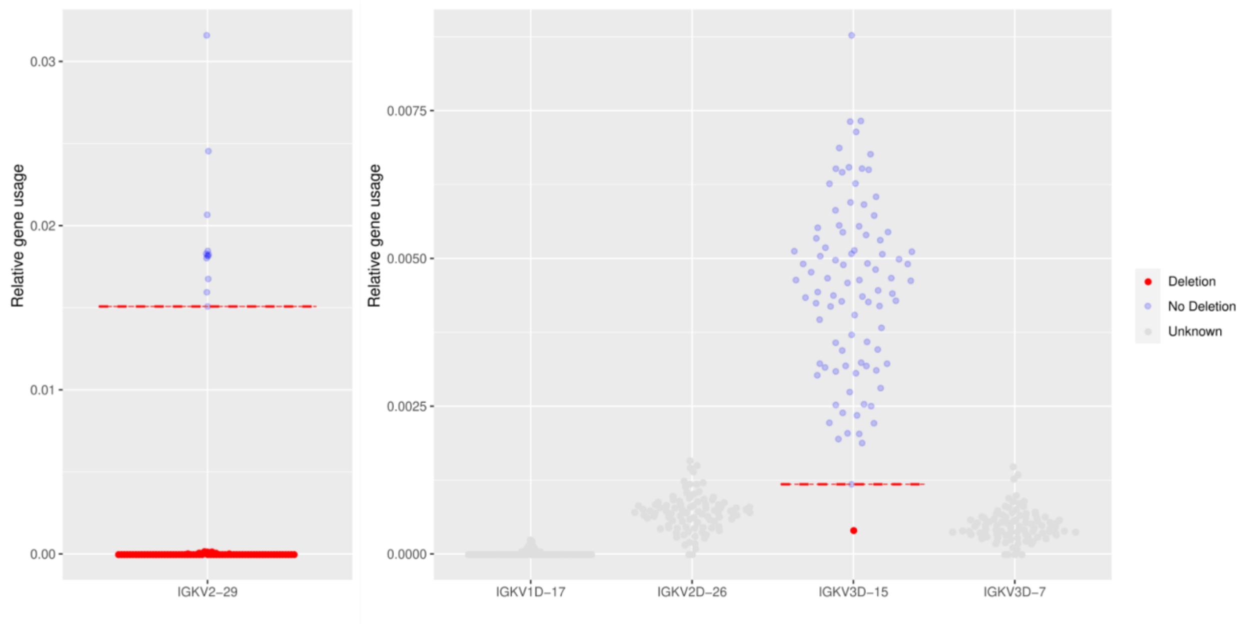

Supplemental Figure S8. IGKV distribution of low expressed genes. Related to Figure 4. The panels in the figure show the relative gene usage. The x-axis shows the different genes and the y-axis shows the fraction of the relative gene usage. Each gene is represented by a box plot, while each individual is represented by a dot. The red line marks the gene specific threshold that was used for the binomial test. The colors represent the presence of a suspected double chromosome deletion, red = deletion, light blue = no deletion, and dark gray = unknown. For usage levels of all functional IGKV genes see Fig.4.
